# Liver stiffness as surrogate parameter in emergency assessment for inpatient health care utilization

**DOI:** 10.1371/journal.pone.0266069

**Published:** 2022-04-08

**Authors:** Dominic Kaddu-Mulindwa, Marius von Martial, Angela Thiel-Bodenstaff, Vadim Lesan, Sebastian Ewen, Felix Mahfoud, Frank Lammert, Marcin Krawczyk

**Affiliations:** 1 Department of Medicine I, Saarland University Medical Center, Saarland University, Homburg, Germany; 2 Department of Medicine II, Saarland University Medical Center, Saarland University, Homburg, Germany; 3 Department of Medicine III, Saarland University Medical Center, Saarland University, Homburg, Germany; 4 Hannover Health Science Campus, Hannover Medical School (MHH), Hannover, Germany; 5 Department of General, Laboratory of Metabolic Liver Diseases, Center for Preclinical Research, Transplant and Liver Surgery, Medical University of Warsaw, Warsaw, Poland; Medizinische Fakultat der RWTH Aachen, GERMANY

## Abstract

**Background:**

Transient elastography (TE) allows non-invasive quantification of liver stiffness (LSM) and steatosis (controlled attenuation parameter, CAP). Here we test the feasibility and utility of TE in the emergency department (ED) and investigate whether LSM predicts longer hospitalization and reimbursement for non-elective patients.

**Methods:**

LSM and CAP were determined in prospectively recruited consecutive adult patients admitted to the ED of a tertiary referral center. Patients were stratified according to the 9.1 kPa and 13.0 kPa LSM cut-offs. Elastography measurements were correlated with clinical and outcome parameters, including duration of hospital stay and hospitalization costs.

**Results:**

In 200 ED patients (133 men, age 18 – 97 years), median LSM was 5.5 kPa (2.4 – 69.1 kPa), and median CAP was 252 dB/m (100 – 400 dB/m). In total, 39 patients (19.5%) presented with LSM ≥ 9.1 kPa, and 24 patients (12.0%) presented with LSM ≥ 13.0 kPa. Heart failure (n = 19) was associated with higher LSM (p = 0.045). Patients with LSM ≥ 9.1 kPa were significantly (p < 0.01) more likely to require longer hospitalization than those with lower LSM. Patients with LSM ≥ 13.0 kPa generated significantly (p = 0.001) higher costs as compared to patients with low LSM.

**Conclusions:**

Transient elastography represents an easily accessible screening tool in ED that might help identify patients in need of increased health care resources.

## Introduction

Emergency departments (EDs) are the front line of our health care systems serving as important source of care with permanent access for patients with different medical needs. Over the last decades the number of ED visits increased substantially [1, 2]. Healthcare professionals at triage in the emergency department stratify patients immediately after admission to limit mortality, morbidity, and health care costs. However, in daily clinical practice it is also important to predict patient admissions to hospital units from the ED to manage bed capacity in an optimal way and to identify patients who are in need of available inpatient resources. This improves the ED boarding process and helps inpatient units to plan the length of hospital stay and discharge ahead of time enabling better health care resource management.

In recent years, transient elastography (TE) has emerged as a new and validated tool that allows assessment of liver fibrosis [3]. Liver fibrosis, in turn, is reflected by the liver stiffness measurement (LSM) during TE. In addition, TE allows simultaneous measurement of liver steatosis by controlled attenuation parameter (CAP) [4]. In brief, using an ultrasound transducer probe with a vibrator, a shear wave is applied that propagates at a stiffness-dependent speed through the liver, which attenuates the signal [5, 6]. TE allows risk-free, repeated measurements with high reproducibility of liver stiffness and steatosis [7–9]. Data on the use of TE as an easy manageable tool in the emergency department (ED) however, are lacking. This study aimed to i) test the feasibility and utility of TE in a tertiary ED and ii) investigate whether TE might help to stratify patients regarding length of hospital stay and iii) if TE helps to predict higher health care costs for non-elective patients.

## Patients and methods

### Study design and participants

This prospective study was conducted in the ED of Saarland University Medical Center (Homburg, Germany). Hemodynamically stable patients (Glasgow coma scale > 13) admitted to the ED between 9 AM and 4 PM from December 2018 to May 2019 (three days per week) were considered for participation. Inclusion criteria were age > 18 years and signed written informed consent to participate in the study. Pregnancy and cognitive or emotional inability to sign the informed consent were regarded as exclusion criteria. Furthermore, patients with ascites were excluded. At least two hours of fasting and a maximum of 200 ml of fluid intake [10] were prerequisites for valid TE measurements.

The following parameters were prospectively collected: age, sex, height, weight, medication, presence of liver disease, diabetes mellitus, and alcohol consumption (frequency per week and number of alcoholic beverages per day). Results of TE (LSM in kPa and CAP in dB/m) were recorded as markers of liver stiffness and hepatic fat contents, respectively. Laboratory parameters (including ALT, AST, creatinine and thrombocytes) were documented.

To evaluate the costs of in-hospital stay, the German DRG (G-DRG) system was used [11]. In Germany, the full and partial inpatient services of the 1.592 general (somatic) hospitals are reimbursed via the DRG system in accordance with Section 17b of the Hospital Financing Act. G-DRG is a performance-oriented and flat-rate remuneration system. Patients with minor illnesses are reimbursed less than patients with serious illnesses requiring more complex treatment. Classification into the DRG flat rate per case is computer-aided (using groupers) and is determined in particular by the type of illness (diagnosis), the severity of the illness, and the services (operations and procedures) performed.

The study was approved by the responsible ethics committee (Ethikkommission der Ärztekammer des Saarlandes, 184/18) and conducted in accordance with the Declaration of Helsinki.

### Transient elastography

All study participants were examined by transient elastography (FibroScan^®^, Echosens, Paris), allowing parallel non-invasive measurements of liver stiffness and steatosis. Patients were in supine position; the right arm placed behind the patient’s head, and the right side of the body stretched towards the investigator. The ultrasound probe was placed at the level of the xiphoid process in the middle axillary line. An attempt was made to collect at least ten valid measurements with a maximum of twelve valid measurements with the median determining liver stiffness and fat content. Measurements were regarded as valid if the interquartile range of LSM was ≤ 30% at a success rate ≥ 60% [12]. In addition to the standard M ultrasound probe, the XL probe was available for obese patients. Patients were stratified according to the LSM ≥ 9.1 kPa [13], and LSM ≥ 13.0 kPa [14] cut-offs.

### Statistical analysis

Numerical variables are reported as means ± standard errors of the mean. The categorical data are presented as numbers and frequencies. The distribution of continuous variables was analyzed using the Kolmogorov-Smirnov test. To compare the means of the numerical data, parametric *t*-tests were used. In the case of the categorical variables, the comparison of proportions was done with chi^2^ tests. Correlation analysis was performed using the Spearman ρ correlation coefficient. For univariate analysis, linear regression was used. All hypotheses were tested at a p-value of 0.05. Statistical analysis was performed using SPSS v25.0 (IBM, Ehningen, Germany).

## Results

**Fig 1:**
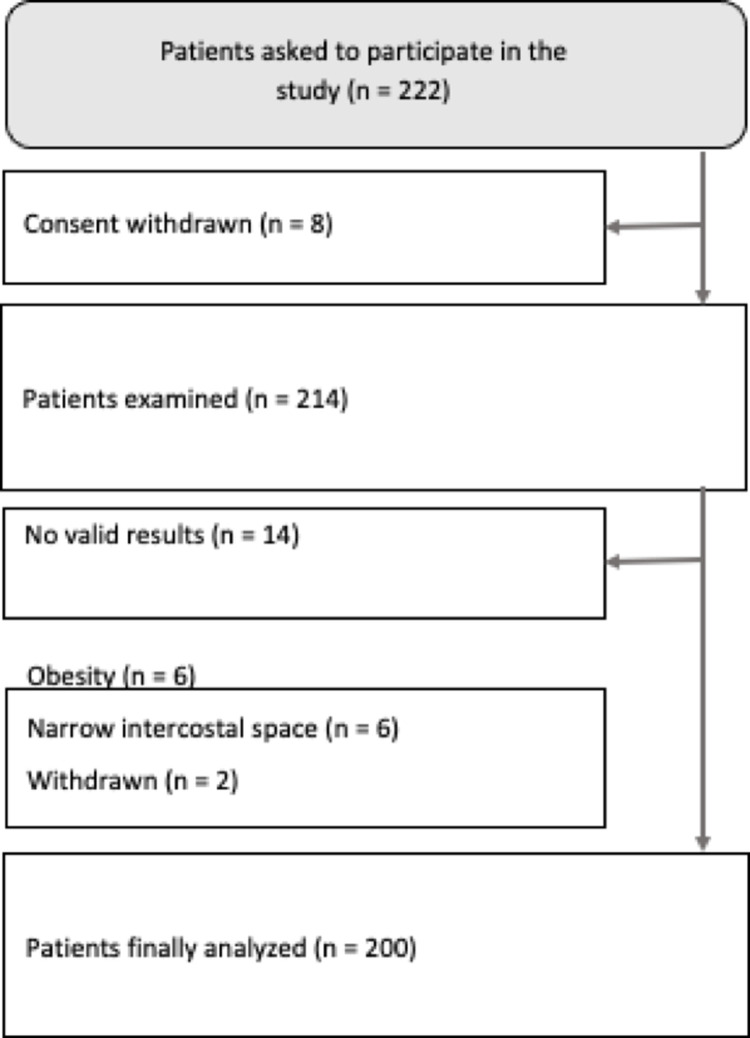
Overview of patients included in this study.

In total, 222 patients were asked to participate in the study, 214 gave their consent (participation rate 96.4%) and were examined at the ED using TE. The mean time for a single examination per patient was 4.0 ± 1.9 min. No valid results could be obtained in 14 patients: in six because of obesity, in six because of narrow intercostal space, and in two patients the examination was stopped prematurely due to withdrawal of patient’s consent (**Fig 1**). Hence, 200 of 214 patients were included in the final analysis. Table 1 summarizes the patient characteristics and laboratory parameters. In brief, the median age was 69 years (range 18 – 97 years), and 33% of the participants were women. Cardiovascular symptoms (39%) and abdominal pain (13%) were the most frequent reasons for ED admission. Among recruited patients, 36 individuals (18%) reported type 2 diabetes and 178 (89%) daily intake of medications. In six patients preexisting liver disease was known based on patients anamnesis (n = 6, five with liver cirrhosis and one with NAFLD), of them 5 had a LSM ≥ 13.0 kPA, respectively meaning that only one patient (with NAFLD) had normal LSM values.

**Table 1:** Clinical characteristics of the study cohort.

Parameters	Subject characteristics
N (women/men)	200 (67/133)
Age (years)	69 (18–97)
BMI (kg/m^2^)	26 (17–39)
Diabetes type 2 (N)	36 (18%)
Regular medication (N)	178 (89%)
ALT (U/l)	24 (7–4,082)
AST (U/l)	28 (14–3,672)
Creatinine (mg/dl)	1.0 (0.2–8.7)
Thrombocytes (10^9/^l)	225 (2–513)
*Period of hospitalization (days)*	
Outpatients (N)	94 (47%)
Inpatients (< 24 hours) (N)	12 (6%)
Inpatients (≥ 24 hours) (N)	94 (47%)
*Alcohol consumption*	
Frequency	
4 or more times a week (N)	17 (8.5%)
2–4 times a week (N)	34 (17.0%)
2–4 times a month (N)	30 (15.0%)
Once a month or less (N)	27 (13.5%)
Never (N)	92 (46.0%)
Number of alcoholic beverages* consumed per day	
10 or more (N)	1 (0.5%)
7–9 (N)	2 (1.0%)
5 or 6 (N)	2 (1.0%)
3 or 4 (N)	10 (5.0%)
1 or 2 (N)	93 (46.5%)
Never (N)	92 (46.0%)
*Sports*	
Several times per week (N)	23 (11.5%)
Occasionally (N)	47 (23.5%)
Never (N)	130 (65.0%)
*TE*	
LSM (kPa)	5.5 (2.4–69.1)
< 9.1 kPa (N)	161 (80.5%)
9.1–12.9 kPa (N)	39 (19.5%)
≥ 13.0 kPa (N)	24 (12.0%)
CAP (dB/m)	252 (100–400)
< 243 dB/m (N)	89 (44.5%)
243–299 dB/m (N)	111 (55.5%)
≥ 300 dB/m (N)	53 (26.5%)

Values are presented as medians and ranges, unless stated otherwise.

*alcoholic beverage corresponds to 0.33 l beer/0.25 l wine/0.02 l spirits, calculated per day of alcohol consumption

Abbreviations: ALT, alanine aminotransferase; AST, aspartate aminotransferase; BMI, body mass index; CAP, controlled attenuation parameter; dB, Decibel; kPa, kiloPascal; LSM, liver stiffness measurement; m, meter; N, number; TE, transient elastography.

As shown in Table 1, median LSM was 5.5 kPa (range 2.4 – 69.1 kPa), and median CAP was 252 dB/m (range 100 – 400 dB/m). In total, 39 patients (19.5%) presented with LSM ≥ 9.1 kPa and LSM ≥ 13.0 kPa was present in 24 patients (12.0%). In addition, 19 patients (8.9%) presented with heart failure, of whom seven showed LSM ≥ 9.1 kPa and six LSM ≥ 13.0 kPa. Massive liver steatosis (i.e., CAP ≥ 300 dB/m) was found in 53 patients (26.5%).

**Fig 2:**
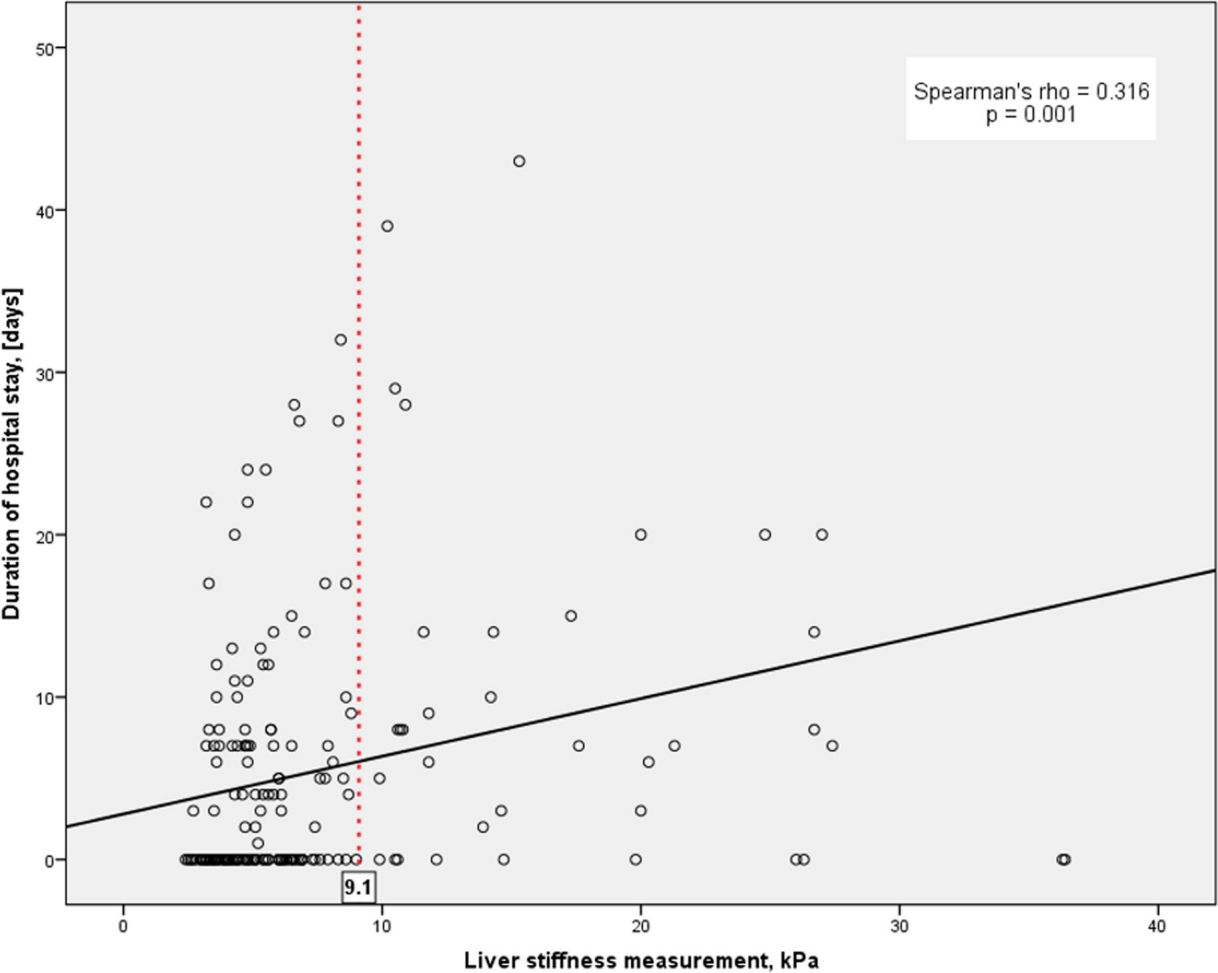
Duration of patient hospitalization in relation to LSM performed at ED.

Table 2 depicts the correlations between LSM and clinical as well as laboratory parameters. LSM correlated significantly with serum aspartate aminotransferase (AST) activity (p = 0.002), creatinine concentrations (p = 0.032), duration of hospital stay (p = 0.001 and **Fig 2**), and health care costs (p = 0.001). CAP, in turn correlated significantly with body mass index (BMI; p = 0.001) and type 2 diabetes (p = 0.03). Table 3 shows that patients with LSM ≥ 9.1 kPa had an odds ratio (OR) of 4.89 (95% confidence interval [CI] 2.27 – 10.50, p < 0.001) for elevated AST activity (> 50 U/l); with LSM ≥ 13.0 kPa this OR increased to 9.19 (95% CI 3.65 – 23.13, p < 0.001).

**Table 2:** Correlation between liver stiffness and clinical and laboratory parameters.

Parameters	Correlation coefficient (Spearman ρ)	p-value
Duration of hospital stay (days)	0.316	0.001
DRG (€)	0.304	0.001
AST (U/I)	0.215	0.002
Creatinine (mg/dl)	0.152	0.032
CLD	0.137	0.053
Type 2 diabetes	0.051	0.475
ALT (IU/I)	0.034	0.630
BMI (kg/m^2^)	0.028	0.694
CAP (dB/m)	0.025	0.728

Abbreviations: ALT, alanine aminotransferase; AST, aspartate aminotransferase; BMI, body mass index; CAP, controlled attenuation parameter; CLD, chronic liver disease; dB, Decibel; DRG, diagnosis related group.

**Table 3:** Odds ratios for clinical and laboratory parameters regarding LSM.

Parameter	LSM ≥ 9.1 kPa			LSM ≥ 13.0 kPa		
	OR	95% CI	p-value	OR	95%CI	p-value
**Male**	0.53	0.23–1.10	0.124	0.79	0.31–2.02	0.632
**BMI ≥ 26 kg/m** ^**2**^	0.90	0.44–1.82	0.789	1.01	0.42–2.37	0.988
**ALT > 50 IU/l**	2.54	1.18–5.49	0.015	3.80	1.56–9.26	0.002
**AST > 50 IU/l**	4.89	2.27–10.50	0.001	9.19	3.65–23.13	0.001
**Creatinine > 1.1 mg/dl**	2.95	1.41–6.16	0.003	2.19	0.91–5.28	0.075

Abbreviations: ALT, alanine aminotransferase; AST, aspartate aminotransferase; BMI, body mass index; kPa, kilopascal.

**Fig 3:**
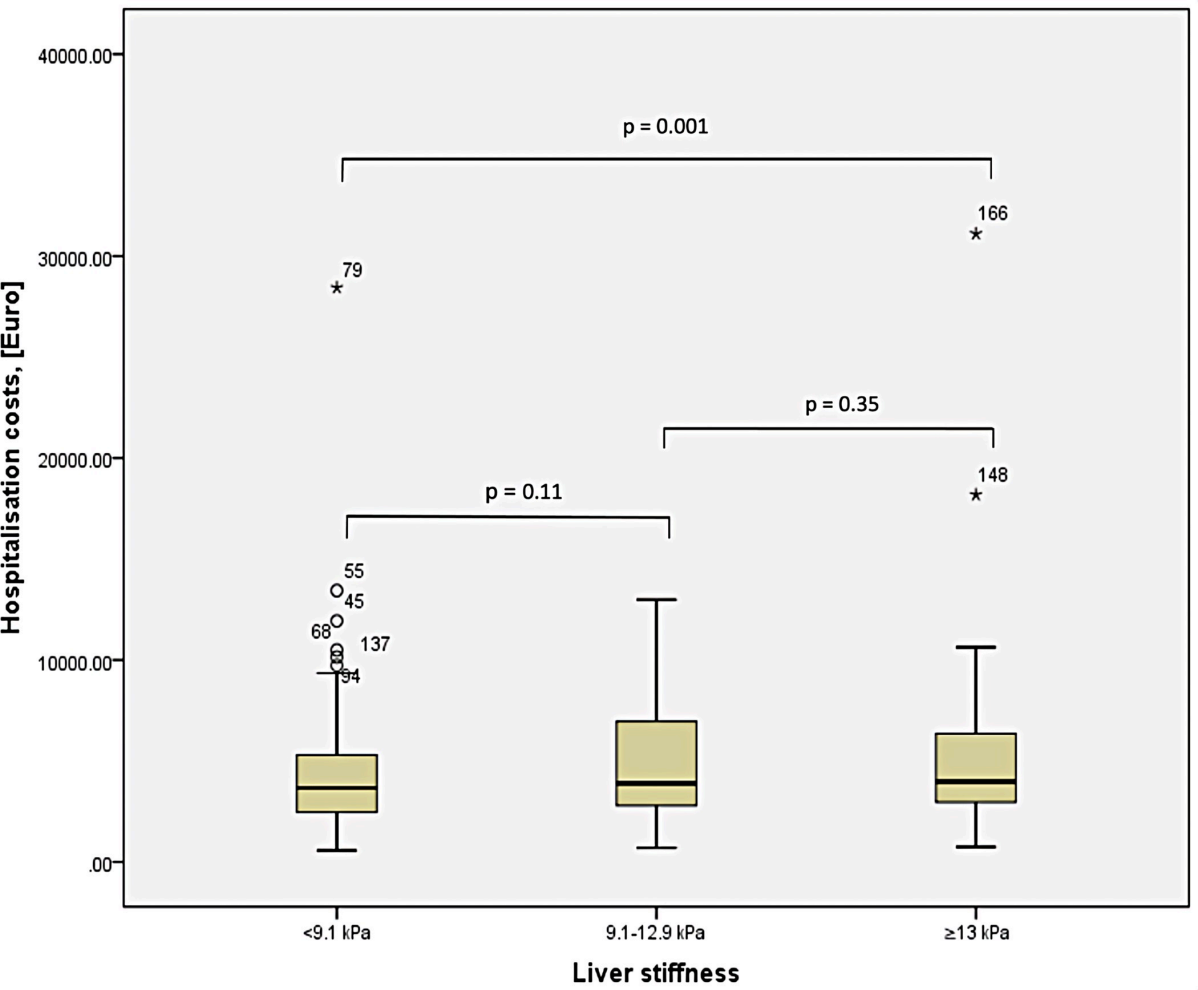
Patient hospitalization costs in relation to LSM performed at ED.

A total of 94 patients (47%) were admitted to specialized departments of Saarland University Medical Center. None of these patients died during the hospital stay. Median duration of hospitalization was 8 days (range 2 – 44 days). As demonstrated in Table 2 and **Fig 2.,** the duration of hospital stay correlated with LSM measured in the ED. In particular, patients with LSM ≥ 9.1 kPa had significantly (p = 0.004) longer duration of hospitalization (mean 11 ± 2 days, range 1–43 days) than those with lower LSM (mean 4 ± 1 days, range 1–38 days). Regarding health care costs, we used the German DRG system (see Methods). The analysis of the entire study cohort demonstrated that patients with LSM ≥ 13.0 kPa generated significantly (p = 0.001) higher costs compared with patients presenting with LSM < 9.1 kPa (5,044.68 ± 1,417.14 *vs*. 2,064.17 ± 271.87 €), as shown in **Fig 3**.

Comparing patients with and without heart failure, we observed a significant (p = 0.045) difference in LSM between both groups: mean LSM in all patients with heart failure was 11.1 ± 8.8 kPa vs. 7.5 ± 7.3 kPa in patients without. Costs reflected by DRGs for patients with and without heart failure did not differ significantly (5,463.39 ± 4,142.70 *vs*. 4,691.03 ± 6,906.06 €).

## Discussion

Over the last years the use of risk stratification models and predictive applications has grown in the healthcare system and have been widely used for decision support in daily clinical practice. To our knowledge, this is the first study that tests the feasibility of TE as an easy manageable tool for stratifying emergency patients in need of more healthcare resources. Additionally, it might serve as a predictor for longer hospitalization and higher health care costs.

Meta-analysis [15] based on 62 cohort studies including in total 43,817 participants with chronic liver diseases showed that higher baseline LSM is associated with an increased risk of all-cause mortality, liver-related mortality, and liver related events during follow-up (by 6–11%). This might explain the longer hospital stays and the higher health care costs incurred by patients with increased LSM albeit there were no hospital deaths in our study. It has to be kept in mind that increased LSM is not exclusively caused by liver diseases but also (right) heart failure and hence, we performed separate analysis of patients with heart failure. We divided our cohort according to the two LSM cut-offs 9.1 kPa and 13 kPa, respectively. Indeed, patients with LSM ≥ 9.1 kPa required longer hospitalization, whereas cases with LSM ≥ 13 kPa generated higher costs. We reckon that the role of TE as a tool that guides patient management decisions should be addressed in future randomized studies.

In line with previous reports [16, 17], we found a correlation between BMI, type 2 diabetes and higher CAP values. Liver stiffness in our ED patients correlated with elevated serum aminotransferase activities and creatinine concentrations and was also a predictor of duration of hospitalization and costs of the in-hospital stay. It has to be kept in mind that even in patients with histologic proven advanced liver fibrosis and cirrhosis serum aminotransferase activities can be normal [18]. Hence, simple blood tests might not be sufficient to detect all patients with liver dysfunction. In this respect, LSM performed in the ED allowed us to detect patients who stay longer in the inpatient units and potentially require more resources during their stay in the hospital. Based on these observations, it is reasonable suggesting that TE might serve as a tool allowing for stratification of subgroups of patients in internal medicine. Furthermore, the data indicates that increased LSM appears to be associated with worse health status, even in the absence of chronic liver diseases. In particular, increased LSM might also indicates heart failure. In our cohort, patients with heart failure had higher LSM compared with those without, which is in line with previously published data [19]. This increase of LSM appears to be caused by higher right-sided filling pressure leading to a stiffer liver [20]. Nevertheless, we found no correlation between higher LSM and longer hospitalization in patients with heart failure, which has been reported previously [21]. One might argue, that TE itself is an expensive tool. Depending on the underlying disease and the local healthcare system structure even one more day of hospitalization generates high costs, therefore predicting longer hospitalization time by TE might justify its high acquisition costs.

Our study has limitations. First, the limited number of patients might have led to an overestimation of the impact of higher LSM on patient hospitalization. In addition, patients who presented directly at the Department of Neurology or were transferred directly to specialized departments were not included in the analysis of the total number of patients presenting at the ED. Second, herein presented results come from a single center, which probably leads to potential confounders due to local resources and the composition of study population underlining the need for further studies as validation of our data. The majority of patients were men, which generally have a higher incidence of liver fibrosis and hence higher liver stiffness in general [22, 23].

In conclusion, our findings demonstrate a high prevalence of advanced steatosis and LSM in adults referred to ED. TE could be an easily accessible and non-invasive diagnostic tool in the ED that might help to identify patients in-need of intensified health care resources. Therefore, it might help to establish better management strategies for patients at-risk with high LSM. Overall, our study could be seen as proof-of-principle for the use of TE in ED. Further longitudinal cohorts with long-term monitoring of patients are needed to confirm these results.
